# Synergy between UCH‐L1 and synaptic loss in Alzheimer's disease retina, predicting cognitive impairment

**DOI:** 10.1002/alz70855_097229

**Published:** 2025-12-23

**Authors:** Altan Rentsendorj, Dieu‐Trang Fuchs, Jean‐Philippe Vit, Edward K Robinson, Alexandre Hutton, Miyah R Davis, Bhakta Prasad Gaire, Stuart Graham, Lon S. S. Schneider, Mehdi Mirzaei, Vivek K Gupta, Keith L Black, Jesse G Meyer, Yosef Koronyo, Maya Koronyo‐Hamaoui

**Affiliations:** ^1^ Department of Neurosurgery, Maxine Dunitz Neurosurgical Research Institute, Cedars‐SInai Medical Center, Los Angeles, CA, USA; ^2^ Department of Neurosurgery, Maxine Dunitz Neurosurgical Research Institute, Cedars‐Sinai Medical Center, Los Angeles, CA, USA; ^3^ Department of Computational Biomedicine, Cedars Sinai Medical Center, Los Angeles, CA, USA; ^4^ Macquarie Medical School, Macquarie University, Sydney, NSW, Australia; ^5^ Alzheimer's Disease Research Center, Keck School of Medicine, University of Southern California, Los Angeles, CA, USA; ^6^ Smidt Heart Institute, Cedars Sinai Medical Center, Los Angeles, CA, USA; ^7^ Advanced Clinical Biosystems Research Institute, Cedars Sinai Medical Center, Los Angeles, CA, USA; ^8^ Department of Neurology, Cedars‐Sinai Medical Center, Los Angeles, CA, USA; ^9^ Department of Biomedical Sciences, Division of Applied Cell Biology and Physiology, Cedars‐Sinai Medical Center, Los Angeles, CA, USA

## Abstract

**Background:**

Alzheimer's disease (AD) pathology extends beyond the brain to the neurosensory retina. Ubiquitin carboxyl‐terminal hydrolase L1 (UCH‐L1), a key enzyme in the ubiquitin‐proteasome system, has a synaptic protective function and was recently implicated in amyloid β‐protein (Aβ) plaque accumulation in AD brains. However, its expression in the retina and potential relationship with synaptic and cognitive integrity remain unexplored.

**Method:**

We used immunohistochemistry and mass spectrometry to assess pre‐ and post‐synaptic markers alongside UCH‐L1 expression in postmortem retinas from AD and mild cognitive impairment (MCI) patients, comparing them to age‐ and sex‐matched cognitively normal controls (NC). Data were correlated with AD‐related brain pathology staging (Braak, ABC scores) and cognitive function (MMSE, CDR scores). An integrated, AI‐based multi‐variable Random Forest analysis identified key predictors of disease status.

**Result:**

MCI and AD patients showed significant retinal synaptic loss in both inner (IPL) and outer (OPL) plexiform layers, with pre‐synaptic markers (VGLUT1: 44%‐59%, Synaptophysin: 56%‐77%) and post‐synaptic markers (PSD95: 44%‐66%, NMDAR2B: 59%‐74%) markers markedly reduced. Membrane‐associated UCH‐L1^(M)^ levels decreased by 43%‐69% across retinal layers. These reductions strongly correlated with retinal Aβ_42_ and phosphorylated tau (pS396, *p* <0.0001) and were closely linked with cognitive dysfunction, higher Braak stage, and ABC scores.

**Conclusion:**

Our findings reveal early and substantial synaptic loss and UCH‐L1^M^ decline in MCI and AD retinas, closely associated with Aβ‐induced p75NTR‐mediated cell death. Random Forest analysis identified UCH‐L1^M^ as a top predictor of brain tauopathy progression (Braak stage) and cognitive impairment (MMSE), highlighting its potential as both a retinal biomarker for AD detection and a therapeutic target.

